# Giant Trichobezoar of Duodenojejunal Flexure: A Rare Entity

**DOI:** 10.4103/1319-3767.65198

**Published:** 2010-07

**Authors:** Mehdi Soufi, Said Benamr, Mehdi Belhassan, Rahal Massrouri, Houria Ouazzani, Bouziane Chad

**Affiliations:** Department of Surgery, B Avicenne, Rabat;; 1 Department of Gastroenterology B, Morocco

**Keywords:** Duodenojejunal flexure, intestinal partial obstruction, surgery, trichobezoar

## Abstract

Bezoars are concretions of undigested material in the gastrointestinal tract, most commonly in the stomach. Duodenojejunal localization of bezoars is exceptional. We report a case of a 27-year-old woman who experienced nausea, vomiting and severe abdominal pain for one week. By palpation a mobile and sensitive mass, 15 × 15 cm, was detected, which filled the upper quadrant. Results of gastric endoscopy were normal. X-ray and ultrasonography suggested a bezoar. A laparotomy revealed that the jejunum was fissured by the trichobezoar ball. This trichobezoar mass was totally excised by intestinal resection.

**Conclusion:**

To our knowledge, this is the first reported case of duodenojejunal fissuration caused by trichobezoar in an adult. Among patients with high subocclusif syndrome, duodenojejunal bezoar should remain a possibility in differential diagnosis.

Bezoars are collections of indigestible material in the gastrointestinal tract. They are of many forms, and the most common forms are phytobezoars, which consist of plant and fruit material; and trichobezoar, which occur from ingestion of hair.

Bezoars have been classified into four types: phytobezoars (caused by vegetables), trichobezoars (caused by hair), lactobezoars (caused by milk curds) and miscellaneous (caused by medications, tissue papers, shellac, tar, sand or fungus).[1]

Trichobezoars are less common than phytobezoars but are more frequently seen in young people and are prevalent in females. Exceptionally they may migrate into the small intestine through the pylorus and become a source of occlusion: this is called Rapunzel syndrome.[1,2]

The trichobezoar, which is an uncommon condition, should be suspected whenever we confront young girls with psychiatric problems who complain about chronic nonspecific digestive problems. The clinical symptoms vary and consist of nonspecific abdominal pain, nausea, vomiting and abdominal mass. Sometimes the bezoar presents itself with gastrointestinal bleeding or occlusion. The endoscopy remains the examination of choice in the diagnosis of intragastric trichobezoar; but in many cases of Rapunzel syndrome, the endoscopic diagnosis is difficult. In these cases, ultrasonography and CT are very helpful for diagnosis. The treatment of trichobezoar is surgical.[1] Endoscopic extraction of bezoar is mostly impossible. Psychological supervision is necessary to avoid recurrence.

Hereby we report a case of duodenojejunal bezoar in a 27-year-old woman presenting with intestinal occlusion which was treated with bowel resection. We want to attract the attention of clinicians to the diagnostic difficulties in this regard.

## CASE REPORT

**Figure 1:**
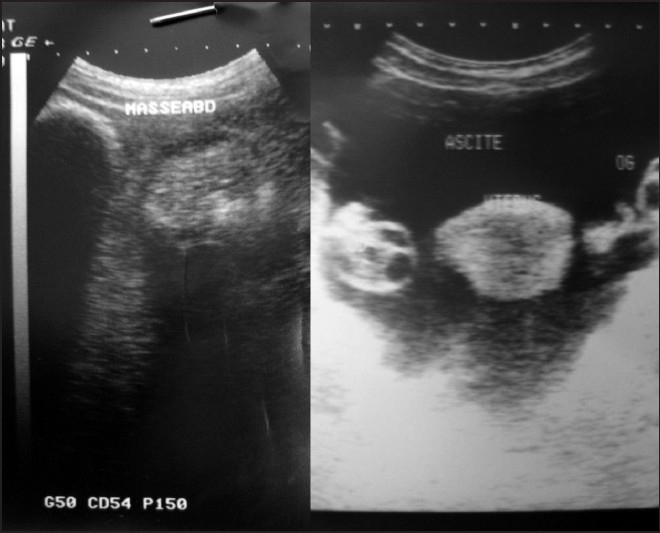
Ultrasonography showing a heterogeneous image in the left upper quadrant and mild ascites

**Figure 2:**
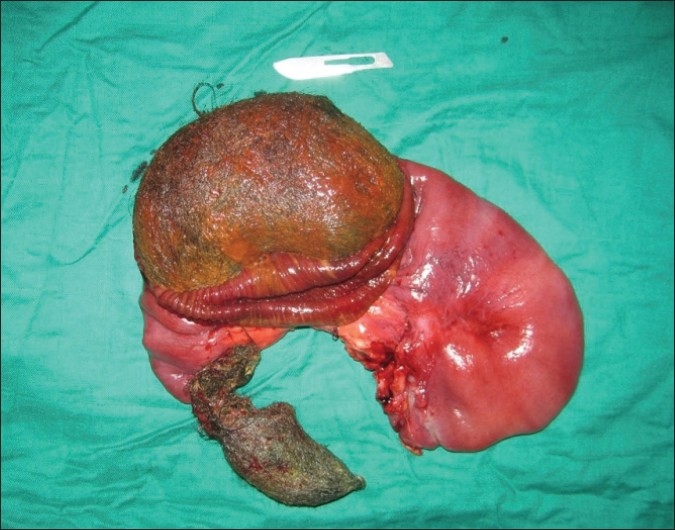
Specimen resection of trichobezoar and the fissured resected small bowel segment

A 27-year-old woman was referred to Avicenne Hospital with a 7-day history of vague abdominal pain and sustained bilious vomiting. Clinical examination showed an oblong mobile and tender mass in the left flank, measuring 10 cm in its greater axis. There was also evidence of paleness of mucosa and skin. Biological examination showed hypochromic microcytic anemia (hemoglobin, 6.2 g/dL), hypoproteinemia (30 g/L) and hypocholesterolemia (1.05 g/L); the rest of biological examination results were normal. The oesogastroduodenal endoscopy did not show any specific anomaly. On the other hand, abdominal x-ray revealed a soft tissue density overlying left upper quadrant. Ultrasonography revealed a heterogeneous image in the left upper quadrant and mild ascites [Figure 1]. A diagnosis of intestinal trichobezoar was suspected. During her hospitalization, the patient developed an occlusive syndrome with severe abdominal pain and vomiting. Clinical examination revealed marked abdominal tympany. The patient underwent urgent operation. The surgical exploration revealed markedly dilated and fissured duodenojejunal flexure with an internal mobile mass. Segmental resection was performed in our patient. After opening the surgical specimen, a trichobezoar measuring 13 × 6 cm appeared in the lumen [Figure 2]. There were no postoperative complications. The patient did not confess having trichotillomania, and psychiatric care was recommended for her.

## DISCUSSION

The term *bezoar* is derived from the Persian word *panzehar*; or the Arabic word *badzehar*, which means masses in the stomach of sacrificed animals used as antidote or antipoison.[1] It is defined as a rare condition secondary to unusual accumulation of substances of different form forming solid masses or concretions inside the digestive tube, mostly in the stomach and sometimes in the small intestine.

Trichobezoars are composed of hair, fur or fibers. This pathology is observed almost exclusively in young girls (90%) aged less than 30 years, complaining of trichotillomania and trichophagia.[1] Despite no reported underlying psychiatric troubles in some cases in the literature, some environmental and psychological factors such as a fragile personality, relational nonadaptation, parents, and depressive states may predispose individuals to this condition.[1,3] Trichobezoar may also occur in patients with digestive antecedents, as in pyloroantrectomy or esophagoplasty.[3]

Rapunzel syndrome was described for the first time by Vaughan *et al*. in 1968.[2] It is a rare form of gastric trichobezoar with duodenal and jejunal extension. (The term Rapunzel-Raiponce in Germany is the name of a heroine with very long hair in the storybook of Grimm brothers.)[3] Like in our patient, clinical picture has no specification; trichobezoar may stay asymptomatic for a long time or may manifest as epigastric discomfort (80%), abdominal pain (70%), nausea and vomiting (65%), asthenia with weight loss (38%) or intestinal transit troubles like diarrhea or constipation (33%).[4,5] Sometimes, the bezoar manifests itself with gastrointestinal complications such as upper gastrointestinal hemorrhage due to ulcerations, mechanical gastric or small intestinal occlusion, gastric or small intestinal perforation with peritonitis or subphrenic abscess, digestive fistula, cholestasis or acute pancreatitis due to obstruction of the ampulla of Vater by the prolongation of the trichobezoar as in Rapunzel syndrome.[6]

In 85% of patients, clinical examination reveals a well-defined abdominal mass that is smooth, firm and mobile in the epigastric area.[5] In our patient, the mass found by examination was due to dilatation of duodenojejunal flexure. Alopecia may also be noted in these patients. Endoscopy remains the examination of choice in the diagnosis of intragastric trichobezoar as it allows visualizing the hair threads, though it wasn’t the case in our patient. A normal oeso-gastroduodenal endoscopy does not exclude diagnosis of jejunal trichobezoar, as reported in many cases of Rapunzel syndrome.[6] The barium follow-through examination shows an intraluminal gastric gap, which is mobile; with a convex border; and in case of a Rapunzel syndrome, there is a duodenal or jejunal extension corresponding to the trichobezoar prolongation.[3] In some cases, abdominal x-ray can suggest the diagnosis by showing heterogeneous density, and abdominal ultrasound would confirm an intraluminal mass with a hyper-echoic arc-like surface and a marked acoustic shadow suggestive of a bezoar.[4,5] These examinations suggested the diagnosis in our patient, and performing CT scan did not seem necessary to us. In other reported cases, CT scan represented the examination of choice. It can delineate a well-defined oval intraluminal mass with air bubbles retained within the interstice or a homogenous mottled appearance in the region of the stomach or intestine.[5] MRI allows making the diagnosis as the mass has variable signals in T1 and T2 and does not take up the contrast after a gadolinium injection.[7] However, these two techniques are expensive and are not essential for the diagnosis of trichobezoar.

Small bowel bezoars are treated surgically. It is mandatory to explore the whole gastrointestinal tract in order to avoid synchronous bezoar and recurrence of intestinal obstruction due to retained bezoar.[8]

The jejunal trichobezoar excision must be done by enterotomy. In our case, resection of the fissured jejunum was necessary.[6,8] Various therapeutic modalities have been proposed to treat trichobezoar and Rapunzel syndrome. Treatment options have been modified with the advent of laparoscopy.[9]

Extraction of mass by endoscopy often fails and may lead to severe complications such as pneumomediastinum due to esophageal fissure. Dissolution of trichobezoar by Papain syrup is ineffective and is not proposed except for phytobezoars. The fragmentation of the ineradicable mass by YAG laser described in 1986[10] constitutes a future perspective.[3,8,10]

Psychological care of trichotillomania and trichophagia is difficult. The patient, as in our report, usually has a refusal attitude, which complicates the diagnosis and management. In cases of associated psychopathological problems such as compulsion, hyperkinetic and depressive syndromes, resort to behavioral treatment becomes necessary.[11]

## CONCLUSION

Rapunzel syndrome is an uncommon trichobezoar, with a tail extending into the small intestine. It has varied presentation and is seldom diagnosed preoperatively. Fissure in duodenojejunal area caused by trichobezoar in an adult patient is uncommon. Duodenojejunal bezoar should be in mind as a possibility in the differential diagnosis of partial intestinal obstruction syndromes.
